# The APPLE Score – A Novel Score for the Prediction of Rhythm Outcomes after *Repeat* Catheter Ablation of Atrial Fibrillation

**DOI:** 10.1371/journal.pone.0169933

**Published:** 2017-01-13

**Authors:** Jelena Kornej, Gerhard Hindricks, Arash Arya, Philipp Sommer, Daniela Husser, Andreas Bollmann

**Affiliations:** Department of Electrophysiology, Heart Center, Leipzig, Germany; Universita degli Studi di Roma La Sapienza, ITALY

## Abstract

**Background:**

Arrhythmia recurrences after catheter ablation occur in up to 50% within one year but their prediction remains challenging. Recently, we developed a novel score for the prediction of rhythm outcomes after single AF ablation demonstrating superiority to other scores. The current study was performed to 1) prove the predictive value of the APPLE score in patients undergoing *repeat* AF ablation and 2) compare it with the CHADS_2_ and CHA_2_DS_2_-VASc scores.

**Methods:**

Rhythm outcome between 3–12 months after AF ablation were documented. The APPLE score (one point for **A**ge >65 years, **P**ersistent AF, im**P**aired eGFR (<60 ml/min/1.73m^2^), **L**A diameter ≥43 mm, **E**F <50%) was calculated in every patient before procedure.

**Results:**

379 consecutive patients from The Leipzig Heart Center AF Ablation Registry (60±10 years, 65% male, 70% paroxysmal AF) undergoing *repeat* AF catheter ablation were included. Arrhythmia recurrences were observed in 133 patients (35%). While the CHADS_2_ (AUC 0.577, p = 0.037) and CHA_2_DS_2_-VASc scores (AUC 0.590, p = 0.015) demonstrated low predictive value, the APPLE score showed better prediction of arrhythmia recurrences (AUC 0.617, p = 0.002) than other scores (both p<0.001). Compared to patients with an APPLE score of 0, the risk (OR) for arrhythmia recurrences was 2.9, 3.0 and 6.0 (all p<0.01) for APPLE scores 1, 2, or ≥3, respectively.

**Conclusions:**

The novel APPLE score is superior to the CHADS_2_ and CHA_2_DS_2_-VASc scores for prediction of rhythm outcomes after *repeat* AF catheter ablation. It may be helpful to identify patients with low, intermediate or high risk for recurrences after *repeat* procedure.

## Introduction

Atrial fibrillation (AF) is the most common clinical arrhythmia associated with significant complications and impaired quality of life. Although medical therapy has limited efficacy compared to invasive AF treatment, arrhythmia recurrences occur in up to 50% of patients within one year after first catheter ablation, while after repeat ablation they still occur in up to 20% [1]. Several observational studies have investigated predictors of arrhythmia recurrence in first procedures. This led to the development of different, partly complicated, scores–e.g. ALARMEc and BASE-AF_2_ –for the prediction of rhythm outcomes following catheter ablation [2, 3]. Some recent studies analyzed predictive value of widely used CHA_2_DS_2_-VASc score for prediction of arrhythmia recurrences after repeat catheter ablation and demonstrated inconsistent results [4, 5].

Based on the results of a previous study [6], we developed and validated a new scoring system for arrhythmia recurrences, i.e. APPLE score, and demonstrated good prediction of arrhythmia recurrences before first ablation [7]. However, whether the APPLE score is useful in prediction of rhythm outcomes in patients following *repeat* catheter ablation is unknown and was analyzed in this study.

## Methods

The study population consisted of patients from The Heart Center Leipzig AF Ablation Registry, Germany undergoing repeat (≥2) ablation according to current guidelines between January 2007 and December 2011. The study was performed according to the Declaration of Helsinki and Institutional Guidelines. Institutional Review Board of Heart Center Leipzig approved the analysis. Patients provided written informed consent. All methods were performed in accordance with the relevant guidelines and regulations.

The APPLE score comprised maximum 5 points (one point for **A**ge >65 years, **P**ersistent AF, im**P**aired eGFR [<60 ml/min/1.73m^2^], **L**eft atrial diameter ≥43 mm, left ventricular **E**jection fraction <50%, range from 0 to 5) and was assessed before procedure. The APPLE score, which is based on clinical variables, is a simple tool with good predictive value and was validated in an external validation set showing similar predictive ability [7].

Mapping and ablation was performed using Ensite NavX, Ensite Velocity (St. Jude Medical, St. Paul, MN, USA) or CARTO 3 (Biosense Webster, Diamond Bar, CA, USA). Trans-septal access and catheter navigation were performed with a steerable sheath (Agilis, St. Jude Medical., St. Paul, MN, USA). A 3D geometry of the LA and the pulmonary veins was obtained and subsequently superimposed on a subtracted 3D-CT or MR-image of the LA. If patients presented with AF, sinus rhythm was restored with electrical cardioversion. In those and in patients presenting with sinus rhythm, completeness of previous antral pulmonary vein isolation and linear lesions was assessed and gaps were closed if necessary. In patients presenting with atrial tachycardia, activation and PPI- mapping and ablation was performed as described previously [8].

After ablation, class I and III antiarrhythmic drugs were not reinitiated and according to the current guidelines [9], oral anticoagulation was prescribed for 3–6 months after catheter ablation and depending on risk stratification of stroke using the CHADS_2_ or CHA_2_DS_2_-VASc score thereafter [10]. All patients were followed for at least 12 months after catheter ablation and 7-day Holter ECG recordings were performed immediately, 3, 6 and 12 months after the ablation. Additional ECGs and Holter ECG recordings were obtained when patients’ symptoms were suggestive of AF. arrhythmia recurrences were defined as any atrial arrhythmia lasting >30 seconds between 3 and 12 months after ablation.

## Statistical Analysis

All statistical analyses were performed with SPSS statistical software version 22 (SPSS Inc., Chicago, USA). Data are presented as means and standard deviation for normally distributed continuous variables and as proportions for categorical variables. The differences between continuous values were assessed using an unpaired two-tailed t-test for normally distributed continuous variables, a Mann–Whitney test for skewed variables, and a chi-square test for nominal variables.

Multivariable logistic regression analysis was performed to identify predictive value of the APPLE, CHADS_2_, CHA_2_DS_2_-VASc scores for arrhythmia recurrences. Receiver operating characteristic (ROC) curves were generated for the analysis of CHADS_2_, CHA_2_DS_2_-VASc and APPLE scores’ performance in predicting rhythm outcomes, with the area under the curve being equivalent to the c-index for determining the predictive value for a score.

A two-tailed p-value <0.05 was considered as statistically significant. All statistical analyses were performed with SPSS statistical software version 22.

## Results

Three hundred and seventy nine consecutive patients undergoing repeat AF catheter ablation were included. The clinical characteristics of study population are presented in **Table 1**.

**Table 1:** Clinical characteristics of the study population.

Variables	Study population	Arrhythmia recurrences		
	n = 379	No (n = 246)	Yes (n = 133)	*p*-value
Age, years	60 ± 10	60 ± 10	59 ± 10	0.483
Males, %	66	33	36	0.430
Persistent AF, %	35	29	45	0.002
BMI, kg/m^2^	29 ± 4.8	28 ± 4.5	29 ± 5.2	0.136
eGFR, ml/min/1.73 m^2^	102 ± 32	102 ± 31	102 ± 35	0.904
Hypertension, %	75	74	77	0.526
Diabetes mellitus, %	13	14	12	0.738
LA diameter, mm	43 ± 6	42 ± 6	44 ± 7	0.005
Δ LA diameter, mm	0 (-4 –(+3))	0.5 (-4 –(+3))	0 (-4 –(+3))	0.804
EF, %	60 ± 10	60 ± 9	59 ± 11	0.601
CHADS_2_ score	1.2 ± 0.9	1.1 ± 0.9	1.3± 1.0	0.052
CHA_2_DS_2_-VASc score	2.0 ± 1.4	2.0 ± 1.3	2.1 ± 1.5	0.218
APPLE score	1.4 ± 1.0	1.2 ± 1.0	1.6 ± 1.0	0.001

**Abbreviations**: BMI–body mass index, eGFR–estimated glomerular filtration rate, LA–left atrial, Δ LA–changes in LA diameter before first and repeat procedure, EF–ejection fraction.

At the time of re-ablation, there were 194 patients (52%) in SR, 98 (26%) with AF and 87 (23%) with atrial tachycardia’s (AT). 57 patients (15%) had completely isolated pulmonary veins. Of 57 patients presenting to repeat catheter ablation with isolated pulmonary veins, 19 patients (33%) were in SR, 13 (23%) had AF and 25 (44%) AT. Generally, arrhythmia recurrences were observed in 133 patients (35%). There was no association between arrhythmia recurrences and age, renal impairment, lower EF or LA changes between the first and repeat catheter ablation. Arrhythmia recurrences occurred in 29% with SR and was 57% in AF (OR 3.212, 95% CI 1.957–5.274 versus SR, p<0.001), and 40% in AT (OR 1.620, 95% CI 0.967–2.715 versus SR, p = 0.067), respectively (p = 0.007).

**Fig 1:**
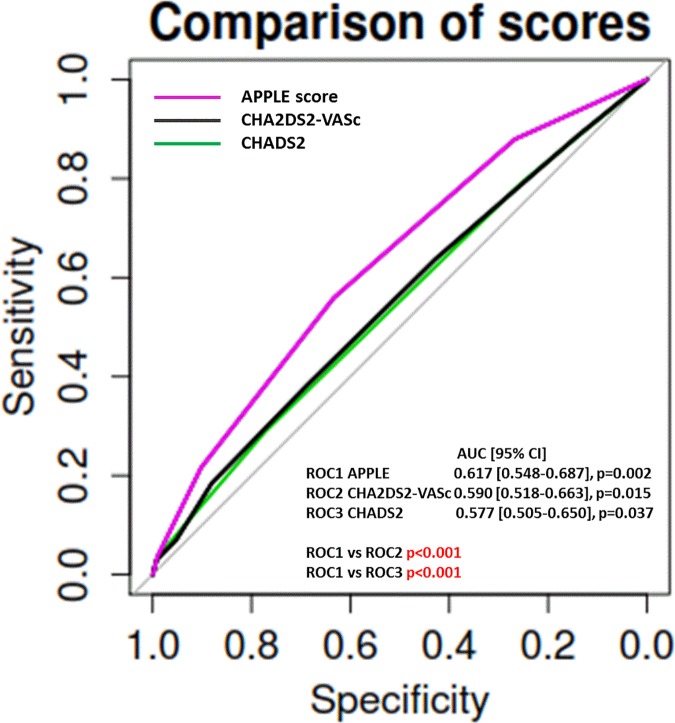
Prediction of arrhythmia recurrences using CHADS_2,_ CHA_2_DS_2_-VASc, and APPLE scores.

While APPLE (OR 1.422, 95% CI 1.155–1.751, p = 0.001) and CHADS_2_ score (OR 1.243, 95% CI 1.005–1.538, p = 0.045) remained significantly associated with arrhythmia recurrences after repeat catheter ablation, CHA_2_DS_2_-VASc score did not (Table 2). Analyzing prediction of arrhythmia recurrences, both CHADS_2_ (AUC 0.577, 95% CI 0.505–0.650, p = 0.037) and CHA_2_DS_2_-VASc (AUC 0.590, 95% CI 0.518–0.663, p = 0.015) scores demonstrated only low predictive value, while the APPLE score ranging from 0 to 5 points, showed significant better prediction (AUC 0.617, 95% CI 0.548–0.687, p = 0.002) compared with other two scores (**Fig 1)**.

**Table 2:** Association with arrhythmia recurrences after repeat catheter ablation using different scores.

Scores	OR	95% CI	*P*-value
APPLE	1.422	1.155–1.751	0.001
CHADS_2_	1.243	1.005–1.538	0.045
CHA_2_DS_2_-VASc	1.094	0.948–1.262	0.218

**Fig 2:**
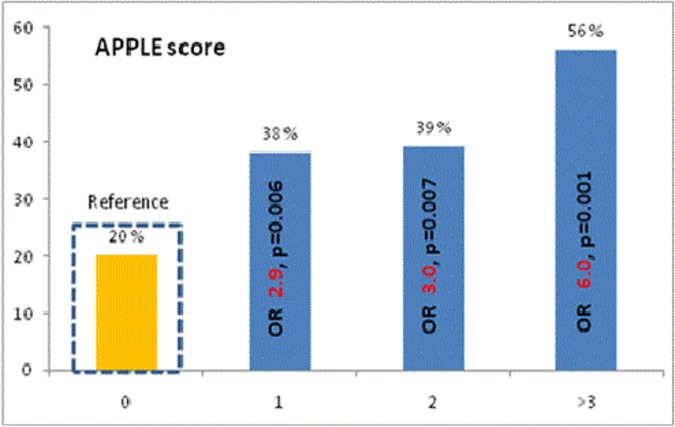
APPLE score and risk for arrythmia recurrences in repeat catheter ablation in whole study population (n = 379). The figure presents incidence of arrhythmia recurrences (%) according to each APPLE score point. Compared to patients with an APPLE score of 0 (reference), the risk (OR) for arrhythmia recurrences was **2.9** (95% CI 1.4–6.3, p = 0.006), **3.0** (95% CI 1.3–6.6, p = 0.007) and **6.0** (95% CI 2.2–16.8, p = 0.001) for APPLE scores 1, 2, or ≥3, respectively.

Patients with APPLE score of 0 (20%), 1 (32%), 2 (23%), and ≥3 (8%) had arrhythmia recurrence rates of 18%, 38%, 39%, and 56%, respectively (p = 0.001, **Fig 2**). Compared to patients with an APPLE score of 0, the risk (OR) for arrhythmia recurrences was 2.9 (95% CI 1.4–6.3, p = 0.006), 3.0 (95% CI 1.3–6.6, p = 0.007) and 6.0 (95% CI 2.2–16.8, p = 0.001) for APPLE scores 1, 2, or ≥3, respectively.

## Discussion

### Main findings

In this study, we demonstrate the predictive value of a new scoring system for the prediction of rhythm outcomes after *repeat* radiofrequency AF catheter ablation in a contemporary AF ablation cohort. Both CHADS2 and APPLE score were significantly associated with arrhythmia recurrences after repeat catheter ablation. However, the APPLE score, which is based on clinical variables, is a novel and simple tool with better predictive value compared to CHADS_2_ and CHA_2_DS_2_-VASc scores.

### APPLE score as predictor for arrhythmia recurrences

Several studies evaluated the predictive value of different scoring systems that were not specifically designed to predict rhythm outcomes after *first* AF ablation (e.g. HATCH, CHADS_2_, CHA_2_DS_2_-VASc scores). A recent large study by Al-Hijji et al [4] failed to demonstrate prediction of arrhythmia recurrences after repeat catheter ablation using CHA_2_DS_2_-VASc score, that is in accordance with our results. Nevertheless, in relatively small cohort of patients with long-standing persistent AF had been recently shown that CHA_2_DS_2_-VASc score ≥3 and renal dysfunction were significantly associated with ablation failure within 31 months [5]. Although, the impact of renal dysfunction on arrhythmia recurrences in patients with first AF ablation had been already shown in our previous research [11], the results of this single center study are difficult to interpret as renal dysfunction (cut off 86 ml/min) was not defined in accordance with current KDIGO guidelines [12].

Of note, two other scores have been developed to predict rhythm outcomes after invasive AF treatment, as ALARMEc (acronym for **A**F type, **L**eft **A**trium size, **R**enal insufficiency, **ME**tabolic syndrome, **c**ardiomyopathy) and **BASE-AF**_**2**_ (acronym for **B**ody mass index >28 kg/m^2^, **A**trial dilatation >40 mm, current **S**moking, **E**arly recurrence, **AF** duration >6 years, **AF** type) [2, 3]. Both scores were developed using much smaller cohorts of patients undergoing cryoablation compared to our initial cohort [6, 7]. Later, Wojcik and co-authors analyzed the predictive value of the ALARMEc score in patients undergoing repeat catheter ablation demonstrating better prediction than CHADS_2_ and CHA_2_DS_2_-VASc scores [3]. Interestingly, between the components of their score, the larger LA size and persistent AF type remained significant predictors for arrhythmia recurrences that was in accordance with our findings, too. Recently, another small study analyzed the predictors for repeat ablation failure in patients with paroxysmal AF and found that changes in LA size was significantly associated with rhythm outcomes [13]. In contrast to these findings, we did not find such association, which might be explained by mixed AF population with both AF types.

### Predictors for arrhythmia recurrences

In a meta-analysis, D’Ascenzo et al [14] demonstrated that persistent AF, LA diameter >50 mm and arrhythmia recurrences within the first month after procedure are the most powerful predictors of AF ablation failure. In contrast to other scores, the APPLE score includes easily obtainable and clearly defined parameters. However, the prediction of arrhythmia recurrences seems to be mostly driven by such components of this score as persistent AF and LA diameter. We did not find significant association between arrhythmia recurrences with age, renal impairment and lower EF. However, it might be explained by 1) small study population, and 2) by young and relatively ‘healthy’ cohort. Our results are in accordance with previous studies and a recent meta-analysis [3, 6, 7, 14]. It seems that the most powerful predictors for the rhythm outcomes remain persistent AF and LA diameter, while other components of APPLE and ALARMEc scores might be considered as possible mediators for arrhythmia occurrence. Nevertheless, using APPLE score it is possible to stratify the risk into different strata that might be helpful for clinical decisions as more aggressive ablation procedure and/or addition of antiarrhythmic drugs within blanking period could be an optimal choice in patients with higher APPLE score. However, the fact that the APPLE score of 0 is still associated with 20% risk of arrhythmia recurrences in patients after *repeat* ablation, indicates that this score needs to be further refined.

### Arrhythmia recurrences and rhythm type before repeat procedure

There are different studies analyzing the association between presenting rhythm and ablation outcome. Ammar et al [15] demonstrated that the freedom from any atrial tachyarrhythmia after *repeat* catheter ablation was reached more often in patients presenting with persistent atrial tachycardia’s than in those with recurrent persistent AF, suggesting that atrial tachycardia’s might be considered as a step toward sinus rhythm. In accordance to these results, we found that persistent AF type at the time of *repeat* procedure was significantly associated with adverse rhythm outcomes. Furthermore, not surprisingly patients presenting with persistent AF had higher risk for later arrhythmia recurrences than patients with sinus rhythm.

## Limitations

This study is limited by its observational, retrospective design. Subgroup analysis of recurrence prediction depending on rhythm (AF vs AT) or ablation strategy especially in patients with isolated PVs was not performed due to small sample sizes. Because arrhythmia recurrences can be asymptomatic and underdetected, further studies with continuous rhythm monitoring during long-term follow-up are needed to confirm our findings.

## Conclusion

Both CHADS2 and APPLE score were significantly associated with arrhythmia recurrences after repeat catheter ablation. Furthermore, the APPLE score is useful to identify patients with low, intermediate or high risk for arrhythmia recurrences after *repeat* procedure. Careful evaluation and stratification of patients before *repeat* procedure using APPLE score may help to identify patients who would profit from repeat invasive AF treatment and improve rhythm outcomes thereafter.
